# Survivin expression in *in situ* and invasive breast cancer relates to COX-2 expression and DCIS recurrence

**DOI:** 10.1038/sj.bjc.6602932

**Published:** 2006-01-17

**Authors:** N Barnes, P Haywood, P Flint, W F Knox, N J Bundred

**Affiliations:** 1 Department of Academic Surgery, South Manchester University Hospital, Manchester, UK; 2 Department of Pathology, South Manchester University Hospital, Manchester, UK

**Keywords:** cyclooxygenase, apoptosis, survivin, breast, DCIS, recurrence

## Abstract

In lung cancer cyclooxygenase-2 (COX-2) expression has been reported to stabilise survivin, an inhibitor of apoptosis (IAP) which prevents cell death by blocking activated caspases. COX-2 expression limits the ubiquitination of survivin, protecting it from degradation. To determine if COX-2 expression in breast cancer showed an association with survivin expression, we assessed the levels of each protein in ductal carcinoma *in situ* (DCIS) and invasive breast cancer (IBC); relating expression patterns to recurrence of DCIS after surgery. Patterns of COX-2 and survivin expression were determined by intensity-graded immunohistochemistry of the primary tumours. Patients with DCIS (*n*=161) which had either recurred (*n*=47) or shown no evidence of recurrence (*n*=114) 5 years following primary surgery were studied. These were compared to 58 cases of IBC. Survivin was expressed in the cytoplasm of 59% of DCIS and 17% of IBC. High levels of both cytoplasmic survivin and COX-2 expression significantly correlated to DCIS recurrence. COX-2 expression was present in 72% of DCIS, and levels of expression positively correlated with cytoplasmic survivin expression in DCIS and invasive disease. The majority of DCIS that recurred expressed both proteins (69%) *vs* 39% nonrecurrent. Recurrence was not seen in DCIS lacking both proteins at 5 years (*P*=0.001). Expression of the IAP survivin is increased in DCIS and correlates closely with COX-2 expression. Increased expression of IAP, (leading to reduced apoptosis) may explain the effect of COX-2 in increasing recurrence of DCIS after surgical treatment.

The incidence of the preinvasive breast cancer ductal carcinoma *in situ* (DCIS) has increased by over five-fold since the introduction of national screening programs (Ernster *et al*, 2000). The recurrence rate in the NSABP B-17 trial at 10 years following breast conserving surgery and radiotherapy remains at almost 12% (Fisher *et al*, 1986). There is, therefore, a need to identify novel predictors of recurrence risk and potential targets for therapy. Cyclooxygenase-2 (COX-2) is an inducible rate-limiting enzyme in the conversion of arachidonic acid to prostaglandins. It is induced by a number of promoters including c-erbB2/(HER2) and NF*κβ* (Smith *et al*, 2000). We have previously shown that COX-2 expression is related to a poor prognostic phenotype of DCIS (Boland *et al*, 2004), but there are no published data on whether COX-2 expression affects recurrence of DCIS. Survivin is a member of the inhibitors of apoptosis protein (IAP) family, which includes proteins such as X-IAP, H-IAP and survivin (Ambrosini *et al*, 1997; Schimmer, 2004), that have effects on both cell cycle regulation and apoptosis. The antiapoptotic properties of the IAP's lie in their ability to block the activation of caspases (mainly 3, 7 and 9) in the cell cytoplasm, which are key regulators of classical apoptosis (Schimmer, 2004). In addition to their antiapoptotic effects, IAP's have been shown to regulate cell cycle progression and cell signalling (Schimmer, 2004). Survivin is expressed in fetal tissues but is absent from normal terminally differentiated cells (Ambrosini *et al*, 1997) (except the basal colon epithelial cells where it may act to limit the stem cell population (Zhang *et al*, 2001)). It is, however, overexpressed in a wide range of human invasive cancers including breast (Tanaka *et al*, 2000), colon (Kawasaki *et al*, 1998), gastric (Lu *et al*, 1998), endometrial (Saitoh *et al*, 1999) and lung (Monzo *et al*, 1999). COX-2 overexpression has been shown to stabilise survivin in nonsmall cell lung cancer by preventing its ubiquitination (Krysan *et al*, 2004) leading to the decreased rates of apoptosis seen in COX-2 overexpressing lung tumours.

The aim of the study was to determine the correlation between COX-2 and survivin expression in DCIS and their relationship to expression levels in invasive disease and recurrence after surgery.

## MATERIAL AND METHODS

### Immunohistochemistry

Archival blocks of formalin-fixed paraffin-embedded tissue were selected, following confirmation that the blocks contained either pure DCIS or invasive breast cancer (IBC) by an experienced breast pathologist (WFK). Staining for estrogen receptor (ER), HER2 and Ki67 and COX-2 have been previously described (Boland *et al*, 2004). For survivin, the slides were dewaxed in xylene and rehydrated through graded alcohols. Following immersion in 0.2% H_2_O_2_ in methanol for 10 min, the slides were placed in citrate buffer for antigen retrieval under pressure. Nonspecific binding was blocked with 10% normal goat serum in phosphate-buffered saline (PBS), with avidin. The primary anti-survivin antibody (Ab469 AbCam, Cambridge UK) was diluted 1 : 400 in normal goat serum/PBS with biotin and incubated on the slides at 4°C overnight. Following further washes in PBS, the biotinylated goat anti-rabbit antibody secondary (BA-1000, Vector, UK) was applied for 1 h. After washing in PBS, the sections were incubated in ABC Vectastain elite reagent (Vector, UK) for 35 min. Staining was visualised with DAB, slides counterstained with 20% haematoxylin, dehydrated, cleared and mounted.

### Evaluation of immunostaining

**Figure 1:**
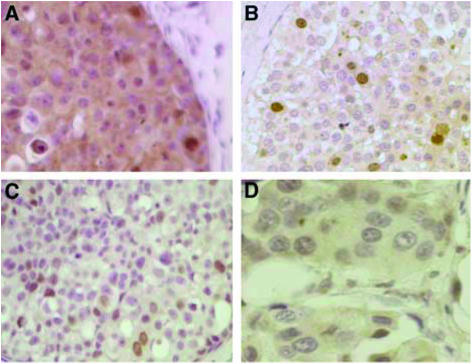
Immunohistochemical staining for surviving. (**A**) Cytoplasmic survivin positive DCIS 3+; (**B**) Cytoplasmic survivin positive DCIS 2+; (**C**) Nuclear survivin alone in DCIS. (**D**) IBC showing cytoplasmic survivin 1+.

Staining was assessed by light microscopy without the knowledge of clinico-pathological features or patient outcome. For ER and Ki67 the percentages of positively staining nuclei were determined. At least 1000 cells were counted for each case, at × 400 magnification. ER status was taken to be positive if ⩾5% of cells were labelled. The membranous and cytoplasmic staining of HER2 was assessed semiquantitatively using the proportion of positively staining cells and degree of staining intensity compared to adjacent normal tissue; samples scored 0 to 3+ as previously described (Boland *et al*, 2004). The cytoplasmic staining of COX-2 was scored 0 (absent) to 3+ (strong) based on the extent and intensity of staining as previously described (Boland *et al*, 2004), COX-2 positivity was defined as a score of ⩾2. Survivin staining was scored for both cytoplasmic and nuclear staining independently. The AbCam ab469 antibody used has been shown to detect all three splice variants of survivin (Fortugno *et al*, 2002) which together are present in both the cytoplasm and the nucleus. Cytoplasmic staining was scored 0 (absent) to 3+ (strong); scores of ⩾2 taken as positive. When cells displayed nuclear survivin the proportion of positive cells out of at least 1000 was determined (Figure 1).

### Patient selection

Women diagnosed with pure DCIS (*n*=161) between 1979 and 1999 were selected, of which, 47 had recurred within 5 years (median time to recurrence 21 months; range 10–60) and 114 were recurrence free after 5 years of follow-up. In all, 13 of the recurrences were invasive disease and 34 were further DCIS. Breast conserving surgery had been performed in 60 of the nonrecurrent and 43 of the recurrent DCIS, and mastectomy in 39 nonrecurrent and eight of the recurrent cases. In our department women with a DCIS exceeding 4 cm would be considered for mastectomy rather than breast conserving surgery. Current policy is to ensure that all wide local excision samples have at least 1 mm clear surgical excision margins. However, this data set used archival specimens, a subset of which did not have clear margins (as they were treated before the benefit of clear margins was proven) therefore, margin status was included in the multivariate analysis of the results. There were not sufficient recurrences following mastectomy to identify significant differences in the nature of recurrence between the mastectomy and breast conserving surgery groups. A random selection of patients with IBC who had undergone surgery between 1980 and 1999 were selected from a tumour bank in our department to compare COX-2 and survivin expression in invasive disease. It was ensured that the invasive samples represented all tumour grades in similar proportions to the DCIS sample. Tumour nuclear grade was determined following review of the original pathology report. Ethical approval was given by the South Manchester University Hospital Ethics Board.

### Statistical analysis

The relationship between receptor coexpression tumour size, nuclear grade, ER status and recurrence were assessed by the *χ*^2^ test. The relationship between Ki67 and receptor expression was assessed using the Kruskal–Wallis test. Correlation coefficients were generated using Spearman's nonparametric correlation. Kaplan–Meier survival plots were generated with log-rank significance. The multivariate analysis was performed using the COX proportional hazards analysis. Significance tests were two-tailed and 5% significance level was used throughout. Analysis was carried out using SPSS 10.0 for windows.

## RESULTS

### Survivin expression in DCIS

In total 102 cases of DCIS were stained for survivin; 69 with no evidence of recurrence at 5 years and 33 with recurrence by 5 years of follow-up. The remaining blocks had insufficient tissue left as COX-2, ER, Ki67 and HER2 staining had already been performed. Survivin was present in both the cytoplasmic and nuclear subcellular compartments. In all, 32 cases (31%) showed no survivin staining (nuclear or cytoplasmic), 10 of 102 (10%) showed nuclear staining alone, 30 of 102 (29%) showed both cytoplasmic and nuclear staining and 30 of 102 (29%) showed cytoplasmic staining alone. The DCIS that recurred was significantly more likely to express moderate to strong (2 to 3+) levels of cytoplasmic survivin (76%) compared to 37% of nonrecurrent disease (*P*=0.0001) (Table 1). Cytoplasmic survivin expression score correlated with COX-2 expression score (Spearman's correlation coefficient 0.322, *P*=0.001). None of the 10 cases that showed nuclear survivin alone recurred within 5 years (Table 1).

There were no significant associations between cytoplasmic survivin expression and the clinico-pathological factors of age, nuclear grade, pathological tumour size, ER, HER2, HER4 status or proliferation (Ki67) (data not shown).

### COX-2 expression in DCIS

As we have previously demonstrated (Boland *et al*, 2004), COX-2 was highly expressed in the cytoplasm of DCIS cells. Overall, 116 of the 161 cases studied (72%) were COX-2 positive (scores of moderate or strong staining). Greater COX-2 expression was seen in DCIS that recurred than the nonrecurrent cases (*P*=0.010; Table 1). The majority of DCIS that recurred expressed moderate or strong COX-2 (Table 1). Only one of the 47 cases that recurred expressed no COX-2 (2%) and only five (11%) expressed weak COX-2 (Table 1), compared to 16% (18/114) and 18% (21/114) cases of nonrecurrent DCIS expressing none or weak staining, respectively. We have previously demonstrated that COX-2 expression correlated with the Type 1 Tyrosine kinase receptor c-erbB2/HER2 expression (Boland *et al*, 2004) and this association was upheld in this new series of women (Spearman's correlation coefficient 0.271, *P*=0.001).

### Differential survivin staining in DCIS *vs* IBC

To assess the differences between survivin expression between DCIS and IBC a panel of 58 IBC's were stained for survivin and COX-2. The grade variation of the invasive cancer was chosen to be similar to that of the DCIS, with six (10.7%) of the invasive cases being grade 1, 13 (23.2%) grade 2 and 37 (66.1%) grade 3. Overall, survivin was expressed at moderate to strong levels in 10/58 (17%) of IBC, compared to 60 of 102 (60%) of patients with DCIS (*P*=0.0001; Table 1). IBC showed weak staining in a further 23 of 58 (40%) of cases. The distribution of nuclear and cytoplasmic survivin also differed significantly between IBC and DCIS, with a higher proportion of invasive cases showing nuclear staining alone 24 of 58 (41%), compared to 10 of 102 (10%) cases of DCIS. Fewer cases of IBC showed evidence of cytoplasmic survivin expression, both alone (12 *vs* 30%), or with coexisting nuclear survivin (5 *vs* 29%) (Table 1). There was no difference in COX-2 expression, in this dataset, between DCIS and IBC.

The presence of cytoplasmic survivin correlated with COX-2 expression in IBC (correlation coefficient 0.28, *P*=0.004) as it did in DCIS. No IBC expressed survivin without COX-2.

### Cytoplasmic survivin and COX-2 coexpression relates to DCIS recurrence

Overall, 13 (17%) cases of DCIS expressed neither protein, 29 (28%) expressed COX-2 alone, nine (8%) expressed cytoplasmic survivin alone and 51 (50%) expressed both proteins.

Of the cases that recurred 70% (*n*=23) expressed both COX-2 and cytoplasmic survivin. In contrast, none of the cases that did not express either protein (*n*=13) recurred within 5 years (*P*=0.013) (Table 2). Coexpression patterns did not relate to cell proliferation, tumour nuclear-grade, ER or HER2 status (Table 2). The relationship between COX-2, survivin and DCIS recurrence was upheld when looking only at the cases that had undergone breast conserving surgery (*P*=0.021) (Table 2).

**Figure 2:**
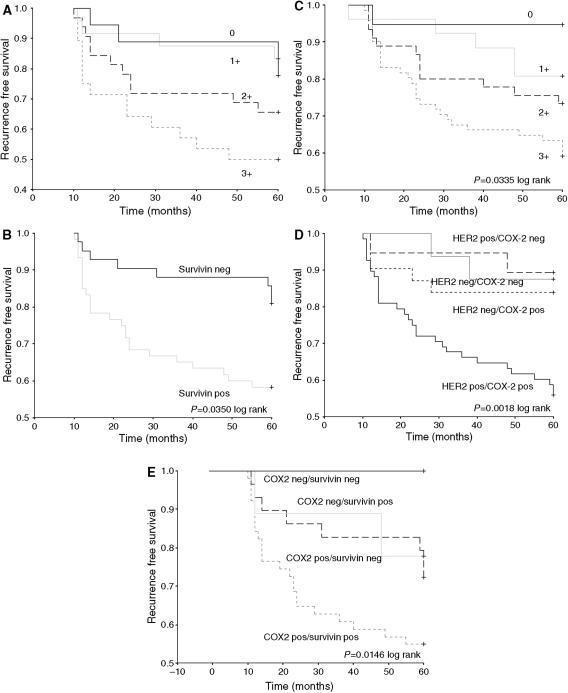
Higher expression of both survivin (**A** and **B**) and COX-2 (**C**) were associated with increased cumulative 5-year recurrence. DCIS that expressed both COX-2 and HER2 (**D**) or COX-2 and survivin (**E**) had the poorest 5-year recurrence-free survival.

When the 5-year cumulative disease free survival was plotted against cytoplasmic survivin status as Kaplan–Meier graphs, DCIS that was cytoplasmic survivin positive (scores of 2 and 3+) had a significantly poorer 5-year outcome than cytoplasmic survivin negative cases (scores of 0 and 1+) (Figure 2A and B). The number of DCIS cases recurring by 5 years also increased with increasing COX-2 score (Figure 2C). We have shown that COX-2 expression is related to the Type 1 Tyrosine kinase receptor HER2 expression (Boland *et al*, 2004). From the Kaplan Meier plot for COX-2 and HER2 expression, it is the tumours that express both factors that have a significantly poorer recurrence-free survival advantage over any other group (Figure 2D).

On combining COX-2 and cytoplasmic survivin coexpression (Figure 2E), the cases that express both survivin and COX-2 have the highest 5-year recurrence. Tumours that are both COX-2 and cytoplasmic survivin negative having the greatest disease-free survival advantage; with none of the cases in this series with both receptors negative recurring by 5 years.

In a multivariate analysis, higher cell-proliferation (*P*=0.006; Exp(B)1.034; 95% Confidence interval (CI) 1.01–1.06), higher nuclear-grade (*P*=0.003; Exp(B) 3.899; 95% CI 1.58–9.61) and higher COX-2 score (*P*=0.019; Exp(B) 1.632; 95% CI 1.09–2.46) were all independent predictors of increased recurrence of DCIS (also included in the analysis were surgical margin status (*P*=0.700), HER2 status (*P*=0.131) and age at diagnosis (*P*=0.419)). The addition of survivin did not aid the model as the two factors correlate in expression frequency.

## DISCUSSION

### COX-2 as a predictor of recurrence and therapeutic target

COX-2 expression in DCIS is a potential predictor of recurrence as well as a therapeutic target. Our own data from breast cancer cell lines, indicates that COX-2 expression inhibits apoptosis, and Celecoxib (a COX-2 inhibitor) reduces xenograft growth by increasing cell death (with no effects on cell proliferation) in a nude mouse model (Barnes *et al*, 2004).

Here, we have shown that COX-2 expression is associated with early recurrence of DCIS (within 5 years of surgery) and is usually associated with survivin expression. We have shown that the cytoplasmic expression of survivin correlates with COX-2 expression and DCIS recurrence. In this study, staining was prioritised for the evaluation of COX-2 and survivin, insufficient tissue remained on enough blocks after this staining for the additional evaluation of markers of apoptosis. However, COX-2 expression has previously been demonstrated to correlate with low-apoptotic rates (Liu *et al*, 2001). This supports the hypothesis that the expression of these two factors is linked via the stabilisation of survivin by COX-2 (as found by Krysan *et al*, 2004) in non-small cell lung cancer), leading to the inhibition of apoptosis. The net effect of which would be to increase the longevity of survivin, thus inhibiting the caspase pathway and increasing cell survival.

### Cytoplasmic survivin expression increases recurrence risk

In IBC, previous studies of survivin protein expression range from 60 (Chu *et al*, 2004) to 94% (Ryan *et al*, 2005). Overall, we were able to demonstrate the presence of survivin in 74% of IBC and 73% of DCIS, with DCIS showing stronger expression levels than invasive cancer; indicating that survivin expression may be an early event in the malignant process.

Survivin, when present in the cell cytoplasm, was associated with increased recurrence of DCIS. We found no correlation between nuclear survivin and recurrence risk. This is a logical finding, as when survivin is in the nucleus it is sub-cellularly distinct from the caspases, which it blocks – which are present and function in the cell cytoplasm. Survivin is, however, a nuclear shuttling protein that exists in three splice variants; wild-type survivin, survivin-2B and survivin delta Ex3. The survivin and survivin 2B splice variants are more often found in the cytoplasm, whereas the delta Ex3 is more frequently found in the nucleus (Rodriguez *et al*, 2002). The antibody we used in this study, detects all three splice variants and further work to characterise the impact of each of these splice variants on recurrence risk needs to be undertaken. Full understanding of whether the differing recurrence/cell signalling effects seen with nuclear *vs* cytoplasmic survivin are due to the subcellular location, or the differential functions of the splice variants remains to be seen. Antibodies to the separate splice variants are not yet commercially available for use in immunohistochemistry.

### Predicting recurrence risk

The optimum treatment of DCIS is still controversial. In the USA, many cases of DCIS do not receive radiotherapy after breast conserving surgery (Baxter *et al*, 2004). Prediction of recurrence risk in both DCIS (and invasive cancer), by way of receptor expression profiling may aid decisions about the need for radiotherapy. Panels of robust indicators of recurrence risk (both clinico-pathological and molecular-biological) could potentially be utilised as routine, to direct individual patient treatment. Identifying patients both at high risk of recurrence, who would require close postoperative radiotherapy and also patients with good prognostic factors that may well be able to avoid unnecessary adjuvant radiotherapy or Tamoxifen.

In this study, we have shown for the first time that lack of COX-2 expression is a predictor of a decreased risk of DCIS recurrence, that is, DCIS that recurs is rarely COX-2 negative. The potential prediction of early recurrence is enhanced upon determining the coexpression of COX-2 and cytoplasmic survivin, as no cases that expressed neither protein in this series recurred within 5 years. This study was a retrospective look at a large number of DCIS cases. It would be beneficial to look in a prospective series of patients – such as at randomised trial follow up to validate our findings.

In IBC, no cases expressed survivin without COX-2, but in DCIS, survivin was present in nine of 102 cases (9%), which lacked COX-2. There must, therefore, be alternate pathways through which survivin is stabilised which are independent of COX-2. Alternatively, the ultimate inducer of COX-2 and survivin may be controlled by up stream, potential candidates being the NF*κ*B or HER2/Ras pathways. In DCIS, COX-2 expression correlated with HER2, thus understanding the downstream signalling via Ras to COX-2 and survivin splice variants warrants further study.

### Inhibiting survivin as a therapeutic target

Survivin and other IAP's have been reported to be upregulated in several malignancies, including breast cancer (Kawasaki *et al*, 1998; Lu *et al*, 1998; Monzo *et al*, 1999; Saitoh *et al*, 1999; Tanaka *et al*, 2000), compared to normal tissue. Phase I clinical trials using the Isis/Eli-Lilly novel survivin RNA antisense inhibitor (LY21818308) are underway to exploit this expression.

In summary, the presence of cytoplasmic survivin correlates with COX-2 expression and recurrence. High expression of both proteins relates to an increased risk of recurrence of DCIS. Assessing the levels of these proteins may identify patients, who could avoid radiotherapy after breast conserving surgery for DCIS. Future use of inhibitors to both these proteins either alone or in combination offer potential new anticancer interventions.

## Figures and Tables

**Table 1:** COX-2 and survivin expression by tumor type

	**Invasive cancer**	**Total DCIS**	**Nonrecurrent DCIS**	**DCIS with 5 year recurrence**
*Survivin subcellular staining pattern*				
No staining	24 (41%)	32 (31%)	24 (35%)	8 (24%)
Nuclear only	24 (41%)	10 (10%)	10 (14%)	0 (0%)
Cytoplasmic only	3 (5%)	30 (29%)	17 (25%)	13 (39%)
Nuclear and cytoplasmic	7 (12%)	20 (29%)	19 (28%)	12 (37%)
*P-*value	0.0001		0.045	

*Cytoplasmic survivin score*				
0	25 (43%)	18 (18%)	14 (20%)	4 (12%)
1+	23 (40%)	24 (24%)	20 (29%)	4 (12%)
2+	7 (12%)	32 (31%)	21 (30%)	11 (33%)
3+	3 (5%)	28 (27%)	14 (20%)	14 (42%)
*P*-value	0.0001		0.05	

Cytoplasmic survivin score ⩾2	10 (17%)	60 (59%)	35 (51%)	25 (76%)
*P*-value	0.0001		0.016	

*COX-2 score*				
0	3 (5%)	19 (12%)	18 (15%)	1 (2%)
1+	11 (19%)	26 (16%)	21 (18%)	5 (11%)
2+	20 (34%)	45 (28%)	36 (31%)	12 (26%)
3+	25 (42%)	71 (44%)	32 (36%)	29 (62%)
*P*-value	n/s		0.09	

COX-2 score ⩾2	45 (78%)	116 (72%)	78 (67%)	41 (87%)
*P*-value	n/s		0.08	

NOTE: n/s= not statistically significant.

**Table 2:** COX-2 and cytoplasmic survivin co-expression in DCIS

	**Protein coexpression**				
**COX-2**	−	−	+	+	
**Cytoplasmic Survivin**	−	+	−	+	***P*-value**
Overall number (%)	13 (17)	9 (8)	29 (28)	51 (50)	
Median Ki67 (%) (*n*=102)	8.7	9.1	11.0	14.4	n/s
Range	1.6–33.5	3.4–40.7	1.6–47.2	2.0–61.1	
Low grade (*n*=8) (%)	0 (0)	2 (25)	4 (50)	2 (25)	n/s
Intermediate grade (*n*=33) (%)	8 (24)	2 (6)	8 (24)	15 (45)	
High grade (*n*=60) (%)	4 (7)	5 (8)	17 (28)	34 (57)	
ER negative (*n*=36) (%)	3 (8)	3 (8)	12 (28)	18 (50)	n/s
ER positive (*n*=63) (%)	9 (14)	6 (10)	16 (25)	32 (51)	
HER2 negative (*n*=36) (%)	7 (19)	4 (11)	11 (31)	14 (38)	n/s
HER2 positive (*n*=66) (%)	6 (9)	5 (14)	18 (27)	37 (56)	
Number no recurrence (%) (*n*=69)	13 (19)	7 (10)	21 (30)	28 (41)	0.013
Number recurred (%) (*n*=33)	0 (0)	2 (6)	8 (24)	23 (70)	
					
*Recurrence type*					
DCIS	0	1	7	16	
Invasive	0	1	1	7	
					
*Breast conserving surgery only*					
Number No recurrence (%) (*n*=39)	6 (15)	4 (10)	15 (38)	14 (36)	0.021
Number recurred (%) (*n*=29)	0 (0)	1 (3)	8 (21)	20 (51)	

NOTE: n/s= not significant; (*n*=xx) is the total number of patients assessed for each parameter.
